# Customizable optode attachments to improve hair clearance timing and inclusiveness in functional near-infrared spectroscopy research

**DOI:** 10.1117/1.NPh.11.4.045011

**Published:** 2024-11-25

**Authors:** Seth B. Crawford, Tiffany-Chau Le, Audrey K. Bowden

**Affiliations:** a Vanderbilt University, Biophotonics Center, Nashville, Tennessee, United States; b Vanderbilt University, Department of Biomedical Engineering, Nashville, Tennessee, United States; c Vanderbilt University, Department of Electrical Engineering, Nashville, Tennessee, United States

**Keywords:** functional near-infrared spectroscopy, spectroscopy, through-hair, optode attachment, neuromonitoring

## Abstract

**Significance:**

Functional near-infrared spectroscopy (fNIRS) is a promising alternative to functional magnetic resonance imaging for measuring brain activity, but the presence of hair reduces data quality.

**Aim:**

To improve research efficiency and promote wider subject inclusivity, we developed the “Mini Comb”—an attachment for commercial fNIRS head caps that can rapidly clear hair with a simple twisting motion.

**Approach:**

To test the utility of the Mini Comb on different hair types, we measured the clearance achieved with eight unique sliding leg extrusions on 10 wigged mannequins of various hair characteristics. Following mannequin testing, we recruited a total of 15 participants to evaluate the performance of the Mini Comb as pertains to the time needed to create clearance and the signal quality captured.

**Results:**

The Mini Comb achieves comparable signal-to-noise ratios (SNRs) as standard hair clearance procedures while reducing hair clearance time by nearly 50%. Importantly, group analysis revealed better SNR results when the Mini Comb sliding leg design is matched to hair type, suggesting that consideration of hair type is important when conducting fNIRS studies.

**Conclusions:**

The Mini Comb thus opens the door for the deployment of fNIRS for more widespread, inclusive, and comprehensive neuroimaging studies.

## 1 Introduction

Due to its portability and lower cost, functional near-infrared spectroscopy (fNIRS) is a promising alternative to functional magnetic resonance imaging for measuring brain activity. fNIRS uses optical signals to measure changes in oxy- and deoxyhemoglobin concentrations that infer cognitive activity. Therefore, a clear pathway for light travel to and from the scalp is critical to obtain high-quality fNIRS data and to generate accurate representations of brain activity.^1^ The presence of human hair, particularly darker hair, in the area of sensing interest is a known challenge that can prevent proper entry and sensing of light through the scalp resulting in signal loss and a reduction in signal-to-noise ratio (SNR).^2^^,^^3^ Moreover, hair characteristics, such as thickness, density, color, and curl or coil patterns, influence the extent of fNIRS signal loss and are known to be significantly different among ethnicities.^4^^–^^6^ For example, African–American hair, which is generally darker and possesses greater hair strand thickness and stronger curl and coil patterns,^7^^–^^9^ can lead to high signal loss. Notably, participants from this demographic are often excluded from fNIRS procedures.^4^^,^^6^ The generalizability of fNIRS research requires adequate representation of many biological demographics. As fNIRS researchers seek to include more diverse patient populations in their study—whether by age, ethnicity, or other attributes—they need better tools to image through the hair of differing properties.

Current strategies to enable through-hair fNIRS usage are inconsistent, proprietary, or time-consuming. For example, one approach anecdotally used by researchers requires shuffling the fNIRS device back and forth across the head to promote movement of the optodes through the hair down to the surface of the scalp. Unfortunately, the effectiveness of the shuffling is often inconsistent across optodes.

Some commercial systems incorporate light guides, which are custom-created mechanical overlays, to help move hair out of the way of optodes^10^^,^^11^; however, the high cost of systems incorporating these features limits accessibility for most researchers and does nothing to help improve the quality of systems they may already own. The “brush optode” introduced by Khan et al.^12^ uses fiber optic bundles attached to optodes to navigate through hair and contact the scalp. Brush optodes have been proven to reduce the setup time and improve SNR and have been incorporated into some modern commercial systems, but the cost of the bundles can be cost-prohibitive, and they may be difficult to incorporate into fNIRS systems for which they were not originally designed.^13^

A common approach employed for hair clearance in the absence of light guides is the use of an external tool (e.g., plastic screwdriver) to clear hair before the optodes are attached to the system. The time-intensive nature of this process is a major disadvantage. The manual clearing process can require up to 45 s per optode,^12^ which can add up to 45 min of setup time for a 60-optode system designed for whole-cortex imaging.^14^^,^^15^ Further, although designs exist for electroencephalography neuromonitoring systems,^16^^–^^18^ no current hair-clearing strategies for fNIRS are designed to create clearance across hair of many different characteristics. Unfortunately, the aforementioned strategies are not inherently customizable, which limits hair-clearing results across diverse patient populations.

In this paper, we first demonstrate our work in designing a novel, three-dimensional (3D)-printed mechanical component that can attach directly to current cap-based fNIRS systems and, second, evaluate the design through mannequin and human testing. The attachment, which we call a “Mini Comb,” features comb-like extrusions that can be customized for versatility and incorporates a twisting mechanism that is simple to operate, thereby reducing system setup time. One of our main focuses in developing the Mini Comb was deployability on a wide variety of hair characteristics (including length, shaft thickness, curl or coil, and density) to promote subject inclusivity with the design. A second focus was to ensure the Mini Comb would be viable across numerous commercially available fNIRS systems. Through mannequin and human testing, we assessed the versatility of the design to create clearance across diverse wigged mannequin populations, validated the improvement in timing, and measured SNR in comparison with commonly used methodologies. In this work, we also show the versatility of the Mini Comb, as small modifications to the design can render it suitable for integration with different commercial systems. The Mini Comb is, thus, poised to enable researchers to perform more effective and inclusive fNIRS imaging of diverse patient populations having many different hair types without requiring investment in specialized fNIRS systems.

## 2 Materials and Methods

### 2.1 Mini Comb Design

**Fig. 1:**
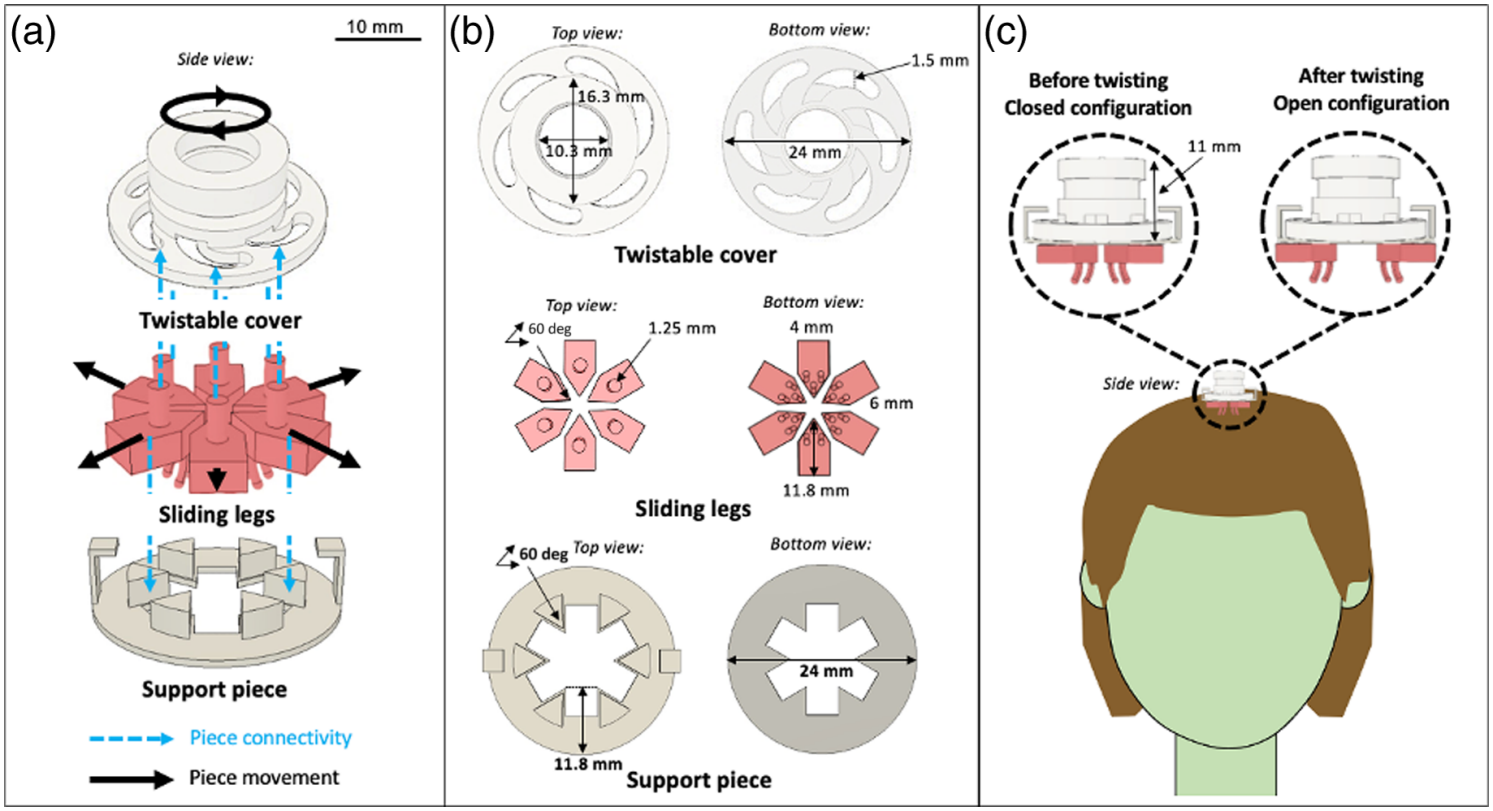
Overview of the Mini Comb. (a) Individual pieces that comprise the total design are the twistable cover (top), sliding legs (middle), and support piece (bottom). (b) Top (left) and bottom (right) views and dimensionality of each piece are also shown. (c) When twisted, the Mini Comp assumes an “open” configuration and clears hair strands from a targeted area on the scalp (Video 1, MP4, 8.39 MB [URL: https://doi.org/10.1117/1.NPh.11.4.045011.s1]).

Taking inspiration from basic claw-shaped hair clips, the Mini Comb is designed to attach to existing cap systems and hold the system’s optodes in place during imaging procedures.^19^ Once attached to the cap, the Mini Comb operates with a clockwise twist (shown in Fig. S1 in the Supplementary Material), which causes the comb-like portion of the design to reposition hair so that the strands are no longer in the optode imaging region. Though the mechanism was conceived to work via a single twist, in practice, improved hair clearance was achieved when the Mini Comb was twisted multiple times (on the order of four twists), meaning repeated twists from an open to closed configuration. Figure 1 shows the overview of its design. The design enables hair clearance in a circular area of the scalp allowing for easy placement of light-emitting or signal-detecting optodes.

The design comprises three parts: a twistable cover, sliding legs, and a support piece. The measurement specifications for the parts can be customized for a given system; the specifications described were designed for use with a Brite MKII fNIRS system (Artinis Medical Systems, Gelderland, Netherlands).

#### 2.1.1 Twistable cover

The twistable cover can be seen in Figs. 1(a) and 1(b) (top). It attaches to an extruded cylinder on the sliding legs via groove-like openings; when the twistable cover is rotated, the groove-like openings convert the rotational movement into linear movement of the sliding legs that allows for hair clearance. The top of the twistable cover is designed as a pocket into which fNIRS optodes can be placed; the inner diameter of the pocket measures 10.3 mm, allowing for optodes of slightly smaller size to securely fit within the Mini Comb. The outer diameter of the twistable cover is 16.3 mm and allows researchers to perform the twisting mechanism. As the Brite MKII system contains cylindrical optodes, similar to most commercial fNIRS systems, the top was designed as a cylindrical disk with an inner diameter slightly larger than that of the optode to enable placement within the Mini Comb. However, by adjusting only the inner diameter of the top on the twistable cover, it can match the optode sizes of other systems.

#### 2.1.2 Sliding legs

The sliding legs, which can be seen in Figs. 1(a) and 1(b) (middle), are positioned radially around the center of the Mini Comb and work in tandem to move hair strands away from the center of the coverage region and to clear a path for the optodes. Through a trial-and-error design process, we determined that six sliding legs are an adequate balance between effectiveness (ability to clear hair) and practicality (ease of printing and assembly).

**Fig. 2:**
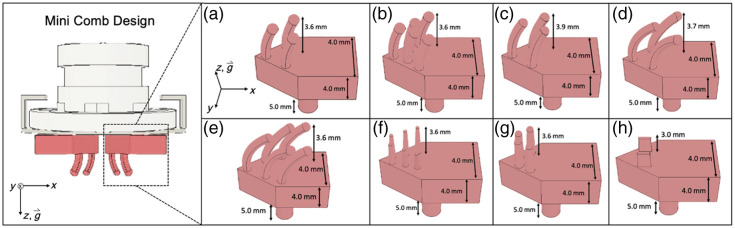
Designs of all comb-like extrusions for sliding legs. The extrusion designs were created for different hair types including straight (a), thick and dense (b), thin and curly [(c) and (d)], thick and curly (e), high level of curl or coil [(f) and (g)], and highly dense (h).

Hair clearance is accomplished through comb-like extrusions, which can be varied to comb through different hair types, present on the underside of the sliding legs that contact the scalp. We tested a total of eight different sliding leg designs (Fig. 2) to accommodate different hair types as characterized by cosmetologists.^16^^,^^20^^,^^21^ The sliding leg designs we selected were motivated by the designs and dimensionality of commonly used combs such as wide-tooth (designs A and C), fine tooth (designs B and E), detangling (design D), Denman (design F), and pick (designs G and H) combs.

The position of each sliding leg is controlled by the twistable cover: the top side of each sliding leg includes a cylindrical post that fits into a slot on the twistable cover. The slot serves as a track to guide the sliding legs between an open (sliding legs apart) or closed (sliding legs together) state.

#### 2.1.3 Support piece

The support piece, which can be seen in Figs. 1(a) and 1(b) (bottom), serves to keep the Mini Comb system connected and in place. The support piece has separators that guide the sliding legs in a linear pathway away from the center of the design. The support piece also contains vertical extrusions that connect it to the twistable cover and keep the whole system connected, which simplifies deployment for researchers.

The height from the top of the twistable cover to the bottom of the support piece is 11 mm [Fig. 1(c)], which is comparable to the height of the current optode holders provided with the Artinis Brite system. Although the height of the entire Mini Comb varies based on the extrusion design of the sliding legs, it is generally <15  mm, allowing both the light emitting and detecting probes (each >15  mm) of the Artinis system to extend through the length of the design and contact the scalp of the participant as originally designed. Because scalp contact can be made, the design does not impede the ability of the fNIRS system to properly couple with the brain.

To demonstrate this versatility of the Mini Comb to fit different continuous wave fNIRS systems, we created additional versions of the Mini Comb that integrate with two other commercial systems: the fNIRS ETG 4000 (Hitachi Philips, Santa Clara, California, United States) and the NIRScout (NIRx, Berlin, Germany) systems. Additional descriptions and figures of these versions are included in Fig. S2 in the Supplementary Material.

### 2.2 Experimental Design

We developed a hair characterization process utilized throughout experimentation on both of our participant types: wigged mannequins and humans. The hair characteristics we considered include length, shaft thickness, curl or coil, density, and color. These characteristics are known to significantly change among adults from various ethnic groups.^4^^–^^9^

To test the Mini Comb design, we followed a similar experimental procedure to what was outlined in the work by Khan et al.^12^ to validate their brush optode design. We collected fNIRS data from human participants and extracted the SNR. However, to optimally match the Mini Comb sliding leg design to human participants, we first conducted wigged mannequin testing. As we cannot collect SNR data from wigged mannequins, we developed a mannequin testing procedure that quantified hair clearance from acquired images. These experimental procedures are described in more detail in Secs. 2.2.1–2.2.3.

#### 2.2.1 Hair characterization

To determine hair length, we collected three strands of hair from the mannequin or participant’s scalp. The three strands were pulled from different locations within the target fNIRS imaging region: one strand was pulled from the medial portion of the scalp (or Cz location according to the international 10–10 optode layout), whereas the other two were pulled from the left (C3) and right (C4) lateral regions of the scalp.^21^ Each strand was collected noninvasively by plucking or cutting at the root. For length measurement, each hair strand was straightened by taping down the ends of the strand to a piece of white paper, and we used a measuring tape to determine the end-to-end length. We calculated the average of the three strand lengths to represent the hair length of the participant.

To determine shaft thickness, we applied a micrometer (VINCA, DMCA-0105) to each of the three hair strands we collected. We ensured the hair strands were not compressed during this process by incrementally tightening the micrometer until the strand could not be removed from the micrometer via gravity. We averaged the three values we collected to record a representative shaft thickness for the participant.

**Fig. 3:**
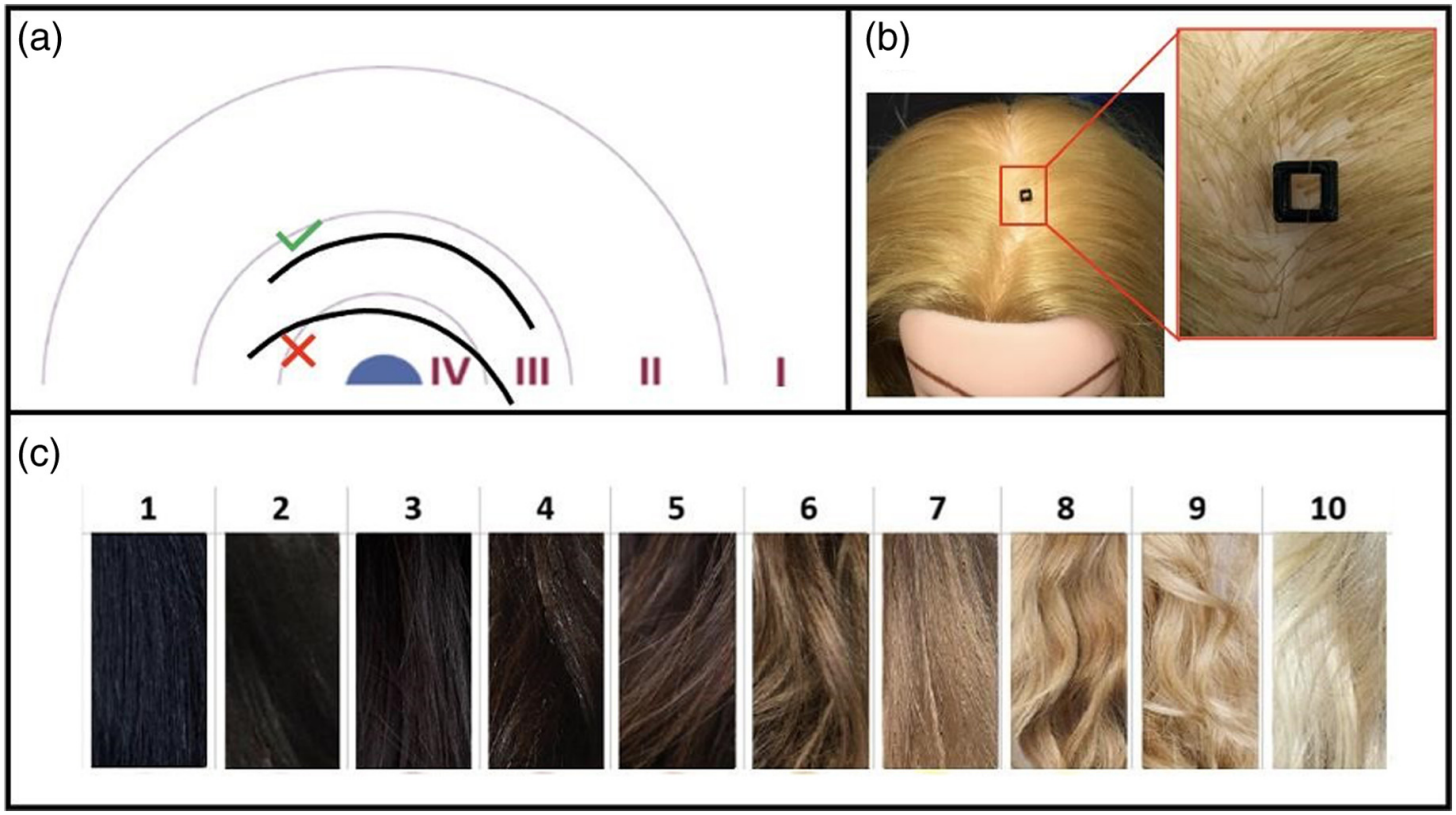
(a) Loussuouarn diagram^20^ used to characterize hair curl or type. (b) Image of 3D-printed window used to determine hair density on a wigged mannequin. (c) Image of color spectrum used to characterize hair color.

To determine the curl or coil^7^^–^^9^ of the hair, we followed a similar procedure outlined by Loussouarn et al.^20^ The Loussouarn group developed and utilized a classification diagram that rates curl on a scale of one to four, which corresponds with a hair type description commonly utilized by cosmetologists.^16^^,^^20^^,^^21^ The diagram contains four curves that correspond to each of the hair types and is shown in Fig. 3(a). To measure curliness, we took a strand of hair and placed it in the area labeled “IV” on the diagram. If the hair strand could not be contained inside of the boundary, it was moved outward to the next area labeled “III.” This movement of the hair was repeated until the hair strand was appropriately contained in a labeled area, and the labeled area corresponded to the representative hair type, which was recorded.

To measure density, we consulted the three locations on the participant’s scalp from which hair strands were collected previously in the procedure. We used hair clips to noninvasively part the hair at each of the three locations and placed a 3D-printed window measuring 3×3  mm onto each part, which is shown in Fig. 3(b). We utilized a macro lens (Xenvo, Clarus 15x) to take an image of the hair contained in the square and manually counted the number of hair strands observed.^12^ We normalized this measurement by recording the density in units of hair strands per square millimeter. We averaged the three hair density values to record a representative value.

To measure color, we utilized a color spectrum and chose the color that blended best with the participant’s hair. We accomplished this visually by comparing the participant’s hair to each color option on the spectrum and recorded the number that matched most closely. The color spectrum is shown in Fig. 3(c).

#### 2.2.2 Mannequin testing

To test the utility of the various extrusion designs and confirm the optimal extrusion design for each hair type, we measured the clearance achieved with eight unique sliding leg extrusions on ten wigged mannequins of various hair characteristics. Wigged mannequins varied in hair length, shaft thickness, curl or coil, density, and hair color, though hair color was not a significant factor in hair clearance on mannequins. To create the wigged mannequin population, we utilized one bald mannequin head and 10 wigs.

The Mini Comb, containing one of the eight sliding leg extrusions (Fig. 2), was incorporated into a commercial fNIRS cap system (Brite MKII) without optodes, which were excluded so that we could evaluate the effectiveness of the Mini Combs for clearance. The cap system was placed on the wigged mannequin head, and the Mini Comb was applied to the C3, Cz, and C4 locations, associated with the international 10–10 optode layout,^22^ then twisted to clear the hair. We chose these optode locations because they are located on the human motor cortex, which is typically a hair-covered region probed during finger-tapping exercises that are commonly used in neuroimaging studies to assess cognitive activity.^12^^,^^23^^–^^25^ At each location, three clearance trials were performed, and the cleared hair was imaged and passed through a MATLAB script (as shown in Fig. S5 in the Supplementary Material). The image was captured with the 8-megapixel main camera on the Samsung Galaxy Tablet A7 Lite and was cropped so that only the 10.3-mm circular area cleared by the Mini Comb was visible. The image was then uploaded to the script that displayed the image and prompted the user to interactively select five pixels on the image that represented the scalp. The image was then converted to grayscale, and the grayscale intensity values at the selected scalp pixels were compiled. The largest intensity value of these pixels then became the intensity threshold in the script, meaning that any pixel with an intensity greater than this value was considered hair, and any pixel with an intensity lower than this value was considered scalp. Each pixel in the image was then binarily identified as either a hair strand or a scalp. The number of nonzero pixels, which represent the scalp, was used to calculate the percent of hair clearance achieved. This process was repeated for all combinations of wigged mannequins and sliding leg extrusions.

#### 2.2.3 Human testing

Following mannequin testing, we recruited a total of 15 participants to partake in two data collection procedures to evaluate the effectiveness of the Mini Comb in two respects: one related to the time needed to create clearance and one related to the signal quality captured with an fNIRS system. The experimental procedure was approved by the Vanderbilt University Institutional Review Board (Vanderbilt IRB#231033). Based on the results from mannequin testing that provided optimal hair clearance effectivity pairs, we determined which sliding leg design was appropriate for each participant.

##### Clearance timing

**Fig. 4:**
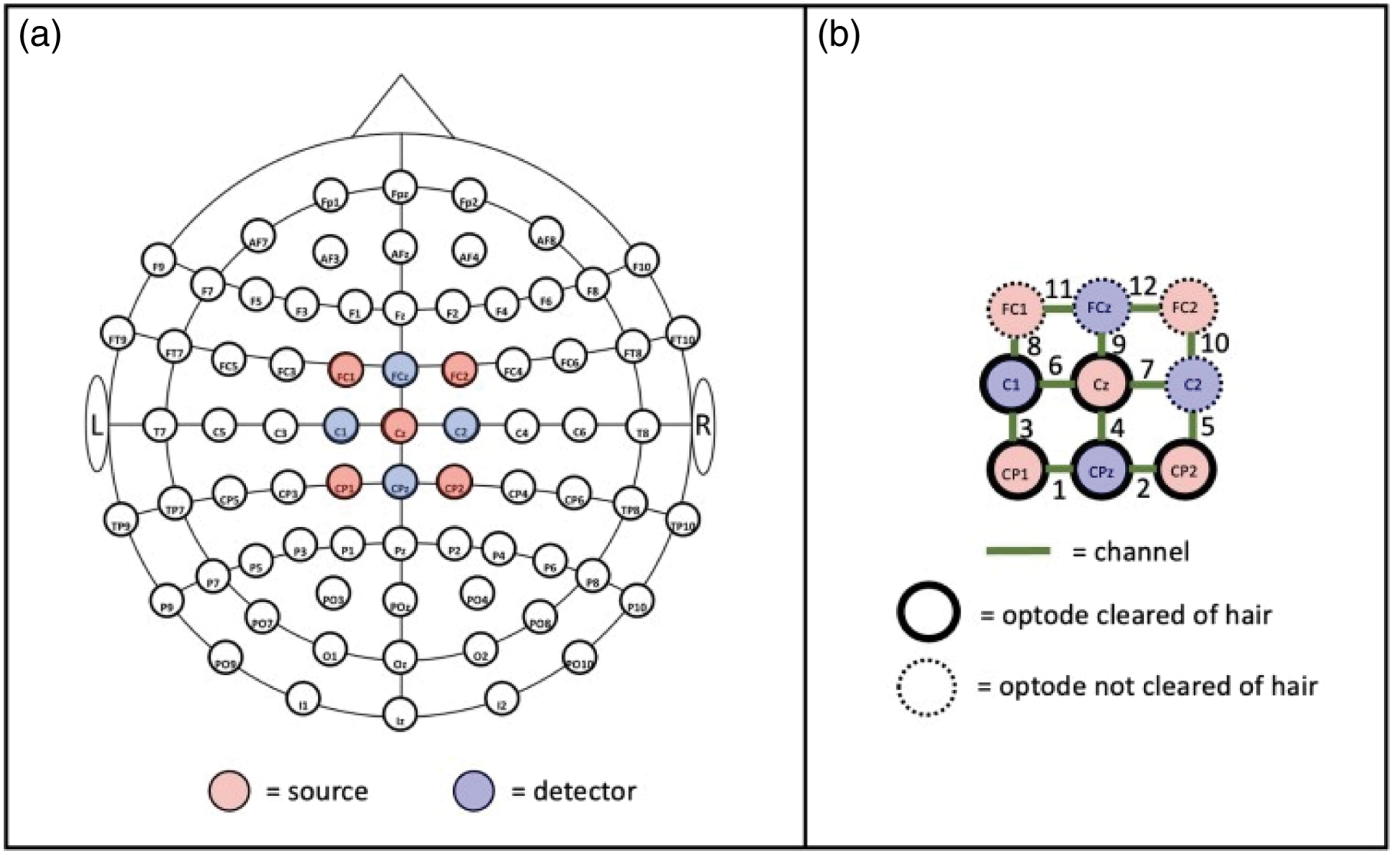
Overview of the fNIRS system arrangement where control and hair-cleared optodes are identified. The optode (a) and channel (b) layout as defined by the 10–10 international layout^26^ for imaging the motor cortex.

The purpose of this procedure was to determine whether the Mini Comb offers an advantage in system setup time over existing methodologies. To perform this validation, we compared the time required to create hair clearance with the Mini Comb and an external tool, a plastic screwdriver, which is typically used to create hair clearance in fNIRS procedures. We placed an fNIRS head cap on each participant’s head and recorded the amount of time needed to create hair clearance in five of nine optode openings across the motor cortex using the plastic screwdriver. We chose this optode split so the remaining four openings could be used as controls during the “fNIRS data collection” procedure. The optode layout is captured in Fig. 4.

We utilized a stopwatch to collect the clearance timing data. Once the fNIRS head cap was properly aligned on each participant, we began the timer and created hair clearance for five optode openings. When creating clearance with the Mini Comb, we repeatedly twisted the design until hair strands were cleared from the inner diameter of the Mini Comb, and the scalp was viewable in each opening. When using the plastic screwdriver, hair strands were moved out of the view of the optode opening again until the scalp was viewable. After this process was completed for each of the five openings, the timer was stopped, and the value was recorded.

We then repeated the cap placement and timing data collection steps a second time for each participant, but in the second trial, we created clearance with the Mini Comb. The hair clearance time (in seconds) recorded for the Mini Comb included the time needed to create clearance in each optode opening via the twisting mechanism. The clearance timings for the Mini Comb and plastic screwdriver were compared using a paired Student’s t-test.

##### fNIRS data collection

The purpose of this procedure was to compare the SNR and signal quality achievable with the use of the Mini Comb compared with the use of the commonly used plastic screwdriver across different hair characteristics. Following each trial of the “clearance timing” procedure, we collected neuroimaging data from the Brite MKII fNIRS system by inserting probes into each of the nine cap openings. The Brite MKII system is a continuous-wave fNIRS system that operates at wavelengths of 760 and 850 nm. Five of the openings were cleared of hair with either the plastic screwdriver or the Mini Comb. As previously mentioned, four openings were not cleared of hair using either method, and we collected data from these openings to serve as a control for the SNR measurements. Once the openings in the cap system were populated with fNIRS system optodes, we collected data over a 7-min time frame consisting of a 2-min initial resting state followed by alternating 40-s stimulus blocks of finger tapping and 35-s rest periods. Once data were collected following hair clearance with the plastic screwdriver and Mini Comb, we extracted the SNR over the four 40-s stimulus blocks of the finger-tapping procedure and computed the average for each block. The SNR was calculated via the following equation where μ represents the signal intensity offset and σ represents the signal variance^27^

$$\mathrm{SNR}=20\log(\frac{\mu }{\sigma }).$$

We performed statistical analysis across the three clearance methodologies using two-way analysis of variance (ANOVA) testing and paired *t*-tests and used Bonferroni correction to address the multiple comparison problem.^28^

## 3 Results

### 3.1 Mannequin Testing

**Fig. 5:**
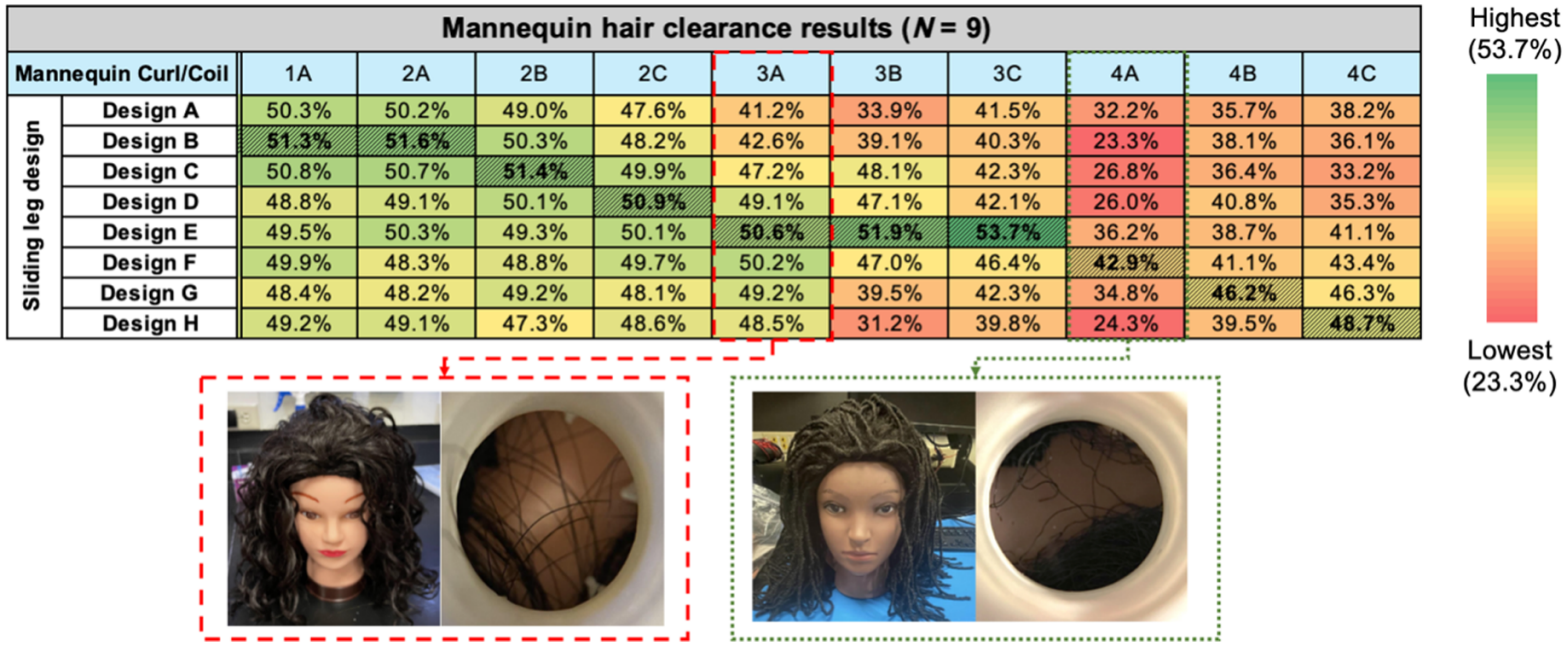
Results from the “mannequin testing” procedure. The quantified results of hair clearance on each wigged mannequin across each sliding leg design are shown in the table. The greatest hair clearance result for each extrusion design is highlighted in the table. Sample images captured with the 8-megapixel main camera on the Samsung Galaxy Tablet A7 Lite show the 3A and 4A wigged mannequins, and a representative picture of the clearance achieved is shown at the bottom.

The results from hair characterization on the wigged mannequins appear in Fig. S3 in the Supplementary Material. The results of the hair clearance tests from “mannequin testing” are shown in Fig. 5. As shown, the clearance variation is very large across different wig curl patterns, so we have highlighted the cell in each column corresponding to the highest clearance value for clarity. As noted, design B worked optimally for the wigged mannequins of hair types 1A and 2A. The dimensionality, spacing, and number of extrusions in design B were inspired by fine-tooth combs, which are often used on hair that has lower density and less curl/coil, such as the 1A and 2A wigs we tested. The tested mannequin with type 1A hair had comparatively low hair density. Typically, straighter hair is associated with greater hair density than hair with more curl/coil,^29^ and this trend is captured in the human participant data collected from the study. In future studies, a more representative wig for type 1A hair will be needed.

Design E was inspired by the fine-tooth comb, but the extrusions were designed with more curvature. We believe this curvature allows for greater interaction with and, thus, more effective clearance in hair with more curl/coil or greater density, such as was the case with the 3A, 3B, and 3C wigs. Designs C and D were inspired by wide-tooth combs, which are commonly used on hair with greater shaft thickness and curl/coil. These designs were found to work optimally on the 2B and 2C wigs as, comparatively, these wigs had higher values of shaft thickness. Designs F, G, and H worked optimally for hair types 4A, 4B, and 4C, respectively. Design F took inspiration from Denman combs, and designs 4B and 4C were inspired by pick combs. These standard comb designs are most often applied to hair with high levels of coil, such as what is seen in wigs 4A to 4C.

### 3.2 Human Testing

**Fig. 6:**
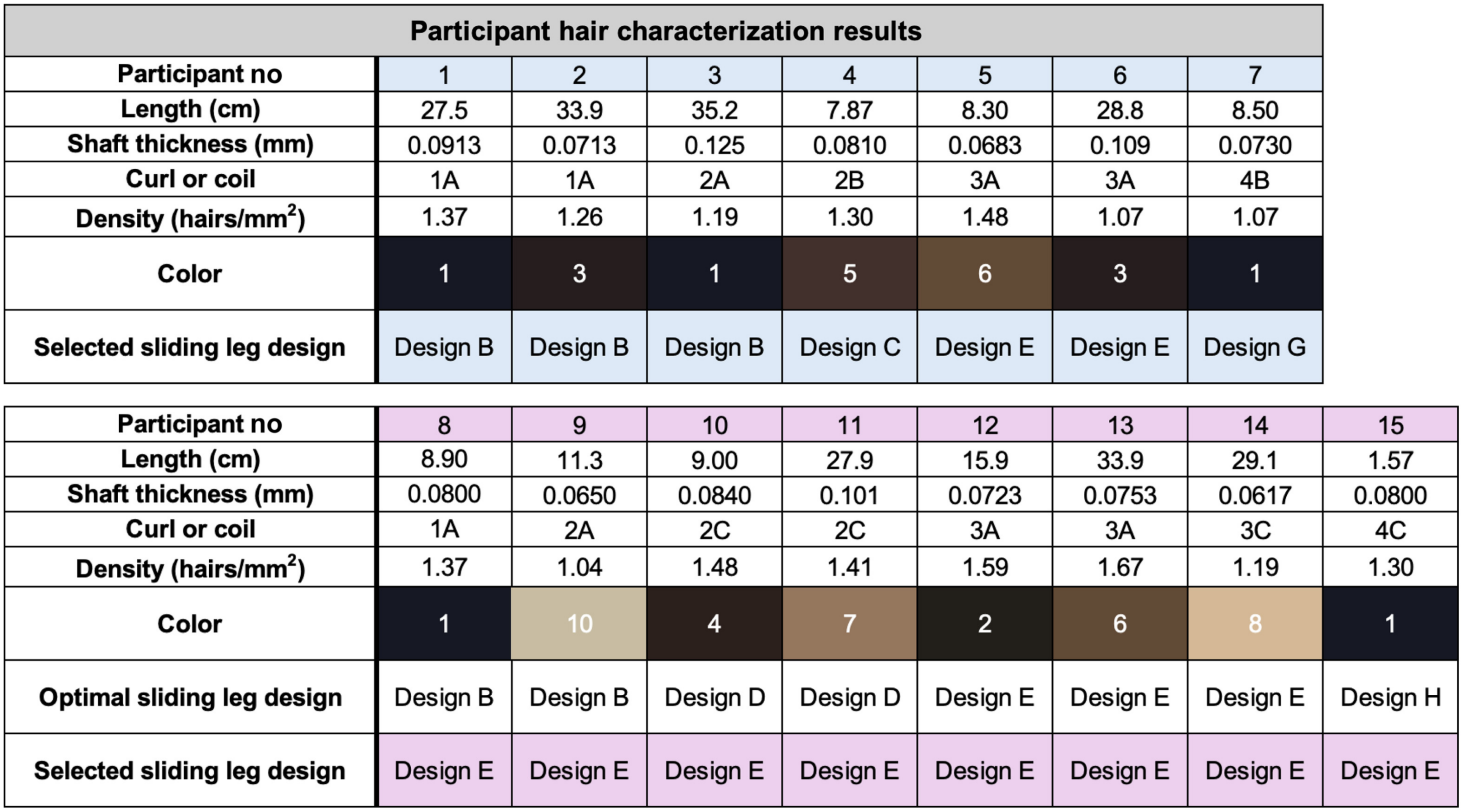
Results from the hair characterization procedure performed on human participants and selected sliding leg design are shown. The last eight participants all used sliding leg design E regardless of hair characterization results.

The results from hair characterization on the human testing participants were compiled and are shown in Fig. 6. We considered and collected data related to color from participants as it is a hair attribute that varies per person and influences fNIRS signal loss; however, hair color did not affect the sliding legs extrusion design chosen for each participant. The main hair characteristic that guided sliding leg design selection was the curl/coil pattern.

We split our participant population into two groups: in the first group, all participants were matched with the optimal sliding leg design, whereas in the second group, all participants were assigned to use sliding leg design E, which was chosen as it showed the highest average level of hair clearance success across all wigged mannequins during “mannequin testing.” This grouping was performed to determine if the choice of the design of the comb-like extrusions influenced hair clearance results.

#### 3.2.1 Clearance timing

##### Findings from group 1

**Fig. 7:**
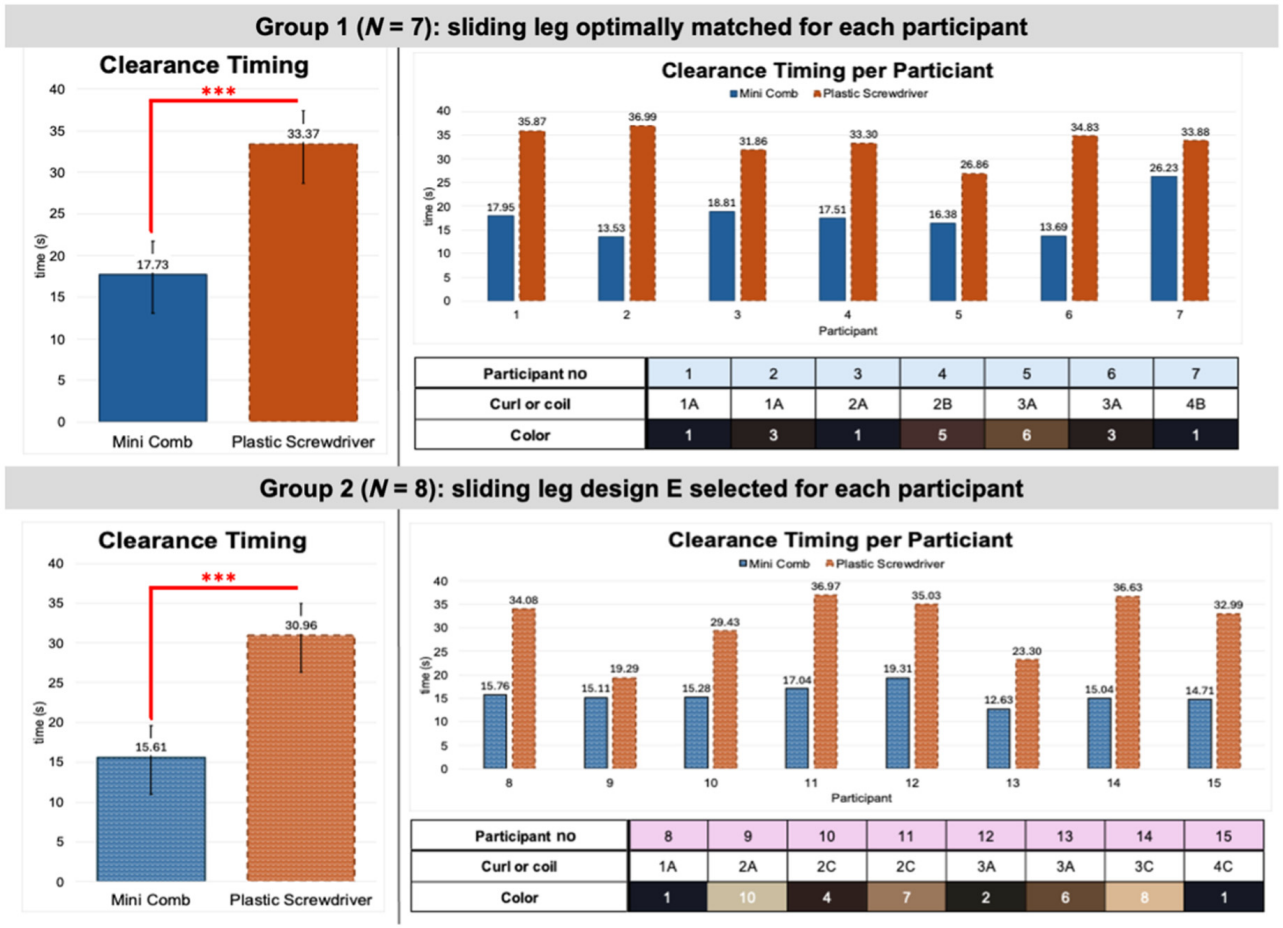
Hair clearance timing data collected across participant group 1, where the sliding leg design was optimally matched to each participant based on mannequin testing, and group 2, where sliding leg design E was chosen for each participant.

Across group 1, which was properly matched with the optimal sliding leg design based on participant hair type or curl/coil patterns, the average time for hair clearance using the plastic screwdriver was 33.4 s, whereas the average time using the Mini Comb was 17.7 s. Using a paired t-test, we determined the Mini Comb significantly (p<0.005) reduced the average time needed to create hair clearance (Fig. 7).

##### Findings from group 2

For group 2, we observed a slight decrease in average timing for both methods, but the Mini Comb (15.6 s) remained significantly lower than the plastic screwdriver (31.0 s) according to a paired *t*-test (p<0.005). The decrease in timing from groups 1 to 2 was likely caused by the researchers growing more accustomed to the hair-clearing process through repetition.

##### Clearance timing improvements with the Mini Comb

Across all participants, the Mini Comb averaged 16.6 s to create hair clearance, whereas the plastic screwdriver required 32.1 s. We found that multiple twists (on the order of four twists), meaning twisting back and forth between fully closed and open configurations, were needed to create proper clearance and move the sliding legs completely out of the path of the system optodes, which increased the average clearance timing for the Mini Comb. In future iterations, we plan to investigate Mini Comb design changes that improve the ease of movement of the Sliding legs to further reduce hair clearance time. Indeed, a robust, one-twist design would reduce the clearance time to under 5 s.

Although the use of the Mini Comb comparatively offers a 15.5-s timing advantage per optode during hair clearance, we found the setup process for the Mini Comb was more time-intensive. For the plastic screwdriver, the setup time comprises the amount of time needed to populate the cap with nine optode holders and position it atop the participant’s head. For the Mini Comb, this setup timing also included the amount of time required to assemble—connect the twistable cover, all sliding legs, and support piece—each of the nine Mini Combs. The average amount of time needed to assemble and incorporate the Mini Combs was 7.1 min, which was significantly longer than the 1.7 min needed to prepare the cap system for use with the plastic screwdriver. Notably, the 5.4-min timing difference across setup and clearance will be overcome using the Mini Comb if a study requires an imaging array with more than 21 optodes (15.5-s timing advantage per optode ×21 optodes = 5.4 min). Also, the steps required for Mini Comb setup could be done prior to participant arrival, ideally via researchers pre-loading the cap system with the Mini Comb sliding leg design of choice. We performed this pre-loading of the cap system for the second group of participants, where the sliding leg design was predetermined and found the Mini Comb setup time decreased to 1.9 min on average.

#### 3.2.2 fNIRS data collection

##### Results from group 1

**Fig. 8:**
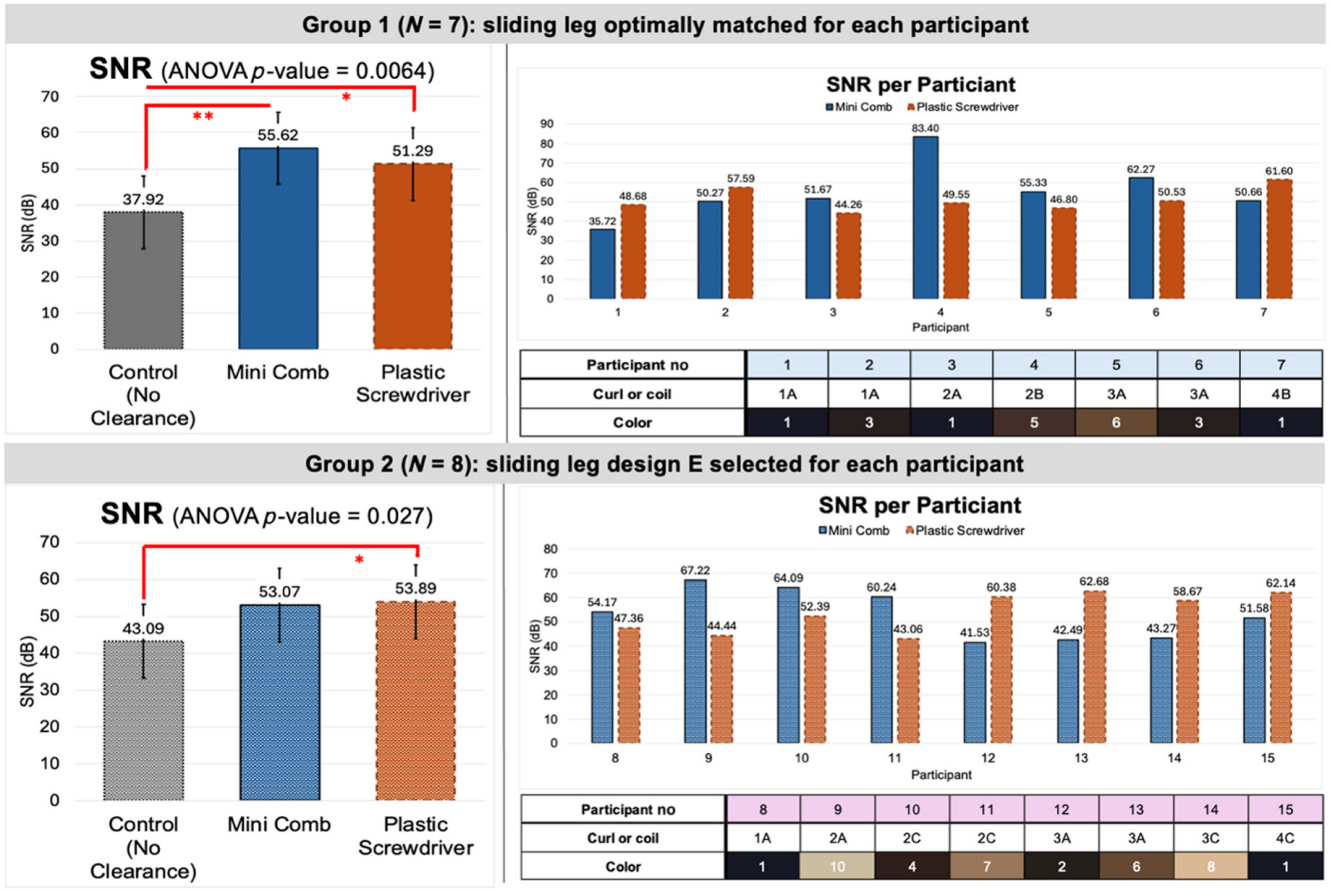
SNR data collected across participant groups 1 and 2.

The average SNRs across group 1 were 55.6, 51.3, and 39.5 dB respectively for channels cleared of hair via the Mini Comb, the plastic screwdriver, and without hair clearance. We conducted ANOVA testing across the results from the three methodologies and found a significant (p<0.01) difference between means. To determine which means are different, we performed paired *t*-tests. We found no significant difference (Fig. 8) in SNR between the Mini Comb and plastic screwdriver (p>0.05), but we did observe that both the Mini Comb (p<0.01) and plastic screwdriver (p<0.05) offered significant improvements in SNR in comparison with the control condition of no clearance.

##### Results from group 2

Across group 2, the average SNR achieved using the Mini Comb with sliding leg design E decreased to a value of 53.0 dB. The SNR for the plastic screwdriver increased to a value of 53.9 dB, and the control condition of no clearance also increased to 43.1 dB. Similar to group 1, we conducted ANOVA testing across the results from the three methodologies (p<0.05) followed by paired *t*-tests to determine the specific means that differed significantly. Using paired *t*-tests, we found no significant difference in SNR between the Mini Comb and plastic screwdriver (p>0.05) nor between the Mini Comb and control condition (p>0.05) when the sliding leg design was not matched to participant hair type. A significant relationship did exist between the plastic screwdriver and the control condition of no clearance (p<0.05) for group 2 participants.

Similar to group 1, the general trends in SNR and hair type existed for group 2. The four participants with the greatest curl/coil characteristics (participants 12 to 15) had the four lowest recorded SNR values with the Mini Comb.

##### Signal quality is improved when the Mini Comb is properly matched to the participants

The increased SNR from groups 1 to 2 when using the plastic screwdriver can be partly attributed to the higher incidence of lighter-haired participants as the average shade of group 2 was approximately two shades lighter based on the hair color diagram used. The increased SNR can also be attributed to the researchers growing more accustomed to the hair clearance procedure as was discussed in the clearance timing results. Types 1A, 2A, and 3A were the only hair types present in both groups, and these hair types had a general increase in SNR from groups 1 to 2 results. Because group 2 data were collected later comparatively, the increased SNR was likely a partial result of the researchers learning how to more efficiently clear hair over the course of data collection.

The decrease in average SNR for the Mini Comb and loss of significance between the Mini Comb SNR and control SNR from groups 1 to 2 can be attributed to the fact that most participants in group 2 were not properly paired to the hypothesized optimal sliding leg design, in comparison with group 1 where all were properly matched based on hair type or curl/coil characteristics. The three participants in group 2 who were truly matched for design E had, on average, longer and denser hair than those in group 1. This supports the hypothesis that a more complex model is needed to match participants with their optimal sliding leg design, rather than using only hair type as the criterion. Nevertheless, the Mini Comb sliding leg design proved to offer similar SNR to existing, common methods for hair clearance while significantly reducing the clearance timing. Notably, a good signal was obtained even in participants with type 4 hair.

## 4 Discussion and Conclusion

In this work, we introduce a novel mechanical attachment called the “Mini Comb” that can connect directly to current cap-based systems to enable rapid clearance of hair with diverse characteristics. The Mini Comb is designed with comb-like features to allow for clearance across different hair characteristics such as thickness, density, color, and curl or coil patterns. With accessibility in mind, the Mini Comb was designed to be easily 3D printed and deployable across multiple continuous wave fNIRS systems—though we believe the system could be iterated in the future to also be deployed with time domain and frequency domain systems. The Mini Comb operates via a twisting mechanism that is simple to operate, thereby reducing the time needed to create hair clearance. The Mini Comb design is comparable to work previously published by Khan et al. that investigated the use of brush optodes for improved signal quality through hair. Similar to the Mini Comb, the brush optodes improved study timing and SNR during finger-tapping exercises; however, we believe the 3D-printable and versatile nature of the Mini Comb design discussed in this work offers a deployability advantage to researchers in comparison with the use of fiber bundles that may be difficult to incorporate into fNIRS systems for which they were not originally designed.

In comparison with using a plastic screwdriver, the Mini Comb was able to clear hair strands from an optode opening at an average of 16.6 s, which is ∼48% faster. Through data collection across 15 participants during a finger-tapping exercise, the Mini Comb produced an average SNR value of 54.3 dB, which was significantly higher than that produced when no hair clearance was created (40.5 dB) and similar to values produced with a commonly used plastic screwdriver (52.6 dB). Thus, the utilization of the Mini Comb produces equivalent signal quality at reduced time in comparison with methods currently used by fNIRS researchers.

Although the results detailed above and the comparison with a common methodology are promising, we have identified some strategies to optimize the hair clearance ability of the Mini Comb. Firstly, we found that additional twists of the Mini Comb incrementally increased the amount of hair clearance created. If the Mini Comb were to be deployed with its current design, we would recommend this strategy of using many twists to accomplish sufficient hair clearance; however, the increase in time needed for these additional twists presents a drawback for this strategy. The need for multiple twists was likely due to imperfections in the design that may be improved through a more detailed study of the supporting legs’ interaction with hair. Hence, another strategy for improving clearance would be to iterate on the current Mini Comb design. The current design was created to make comfortable contact with the participant’s scalp^30^^–^^34^ without causing excess pressure.^35^^,^^36^ Our group plans to explore iterations that would enable a single twist to create better clearance and investigate new sliding leg designs. We hypothesize that sliding leg designs with an increased number of comb-like extrusions and alternative designs at the hair-contacting component of the extrusions, which are currently all spherically shaped, could improve achievable clearance. These iterations are feasible but would require a great time investment, as these changes may introduce challenges in terms of fabrication, 3D printability, and comfort for the participant. Further, although many labs have access to a 3D printer, the resolution of the printer and the time required to produce the number of components needed to create clearance across larger imaging layouts may present problems for some researchers. Therefore, future testing and iterations of the Mini Comb should consider simpler designs that may offer improvements to both the twisting mechanism and fabrication process. Researchers may also consider hiring print services that can offer low-cost options when printing at scale.

To further optimize the Mini Comb’s usability, we recommend preparing the Mini Comb prior to participant arrival; ideally, the researchers could pre-load multiple cap systems with the Mini Combs of different types and quickly select the optimal system for use when the study commences. The dimensions of the support piece in the overall Mini Comb design also limit its ability to be used for short-channel data collection. However, it is reasonable to assume that the design can be tweaked in future iterations to allow for source-detector separation distances below 24 mm.

We also recognize a more thorough investigation of the relationship between signal quality and the Mini Comb is likely required for widespread acceptance of the design. In this study, we used SNR as our indicator of signal quality, but SNR is influenced by other factors such as hair color, as previously discussed, and skin pigmentation in addition to hair clearance. Although skin pigmentation was not a variable that was collected for analysis in this study, we plan to perform future investigations that consider these other important variables to more accurately extract the impact of the Mini Comb.

The SNR we were able to achieve with the Mini Comb was also limited by the choice of sliding leg design used in human testing. The optimal sliding leg was chosen based on performance on wigged mannequins, but these optimal pairing mays have been skewed by differences in the wigs and human hair. As we mentioned previously, at least one of our wigged mannequins had hair density inconsistent with what is typically seen based on the hair type. A more accurate study would use human testing to determine optimal designs for human studies.

Another limitation exists in participant recruitment. We focused our recruitment efforts to ensure we had at least one participant for groups one and two that had hair curl/coil patterns across types 1 to 4. Although this diversity of curl/coil allowed us to test a wide range of sliding leg designs, it did not provide us with the necessary controls to properly match sliding leg designs with human participants. Most participants in the study varied largely from one another across all five measured hair characteristics. To create a proper model for matching optimal sliding leg designs with participants, the ideal experiment would control for individual characteristics. Post hoc testing (Fig. S4 in the Supplementary Material) with two new wigged mannequins both of type 4A hair support this need for a more complex matching procedure as a different sliding leg design created optimal hair clearance for each wig. Future efforts will be dedicated to testing clearance on a larger and more diverse set of hair characteristics, particularly more participants with longer, type 4 hair.

In general, we expect that the Mini Comb will enable researchers to perform rapid hair clearance and more effective and inclusive fNIRS imaging of diverse patient populations without requiring investment in specialized fNIRS systems.

## Supplementary Material





## Data Availability

The data that support the findings of this study are available from the corresponding author upon reasonable request.
