# Supplementary material for: Customizable optode attachments to improve hair clearance timing and inclusiveness in functional near-infrared spectroscopy research
Source: Neurophotonics. 2024 Nov 25;11(4):045011. doi: 10.1117/1.NPh.11.4.045011 (PMC11587899; doi:10.1117/1.NPh.11.4.045011)
Supplement: Supplementary file 1 [file NPh_011_045011_SD001.pdf]

Customizable optode attachments to improve hair clearance timing and inclusiveness in functional near-infrared spectroscopy research

1. Supplementary Material

1.1 Mini Comb design and twisting mechanism

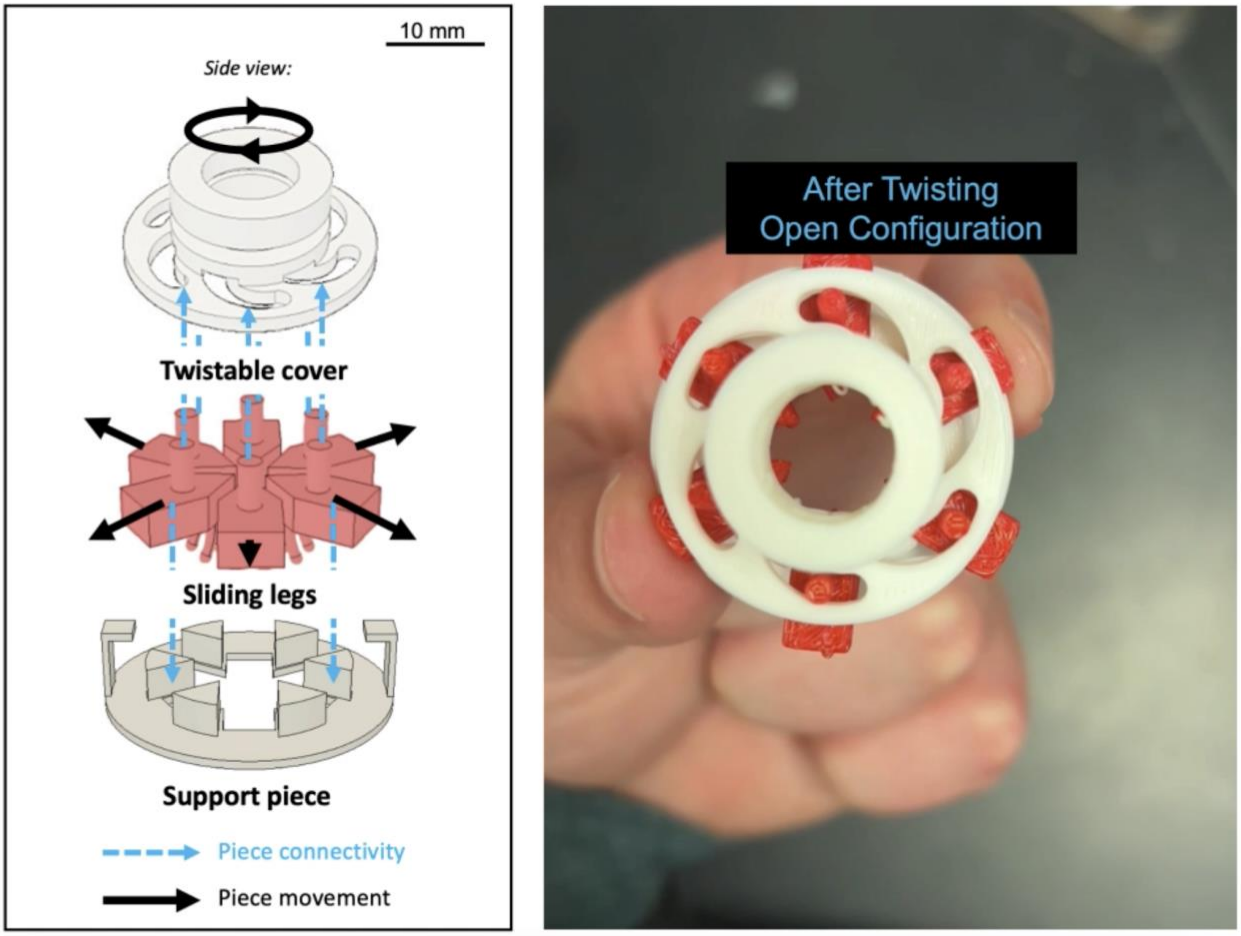

Figure S1. In-hand Mini Comb design and twisting mechanism utilized to create hair clearance (Video 1, MOV, 8.8 MB).

1.2 Design Versatility

To demonstrate this versatility of the Mini Comb to fit different systems, we created additional versions of the Mini Comb that integrate with two other commercial systems: the fNIRS ETG 4000 (Hitachi Philips – Santa Clara, CA) and the NIRScout (NIRx – Berlin, Germany) systems. The optode sizes of both systems were unique and larger than that of the Brite MKII system. The Mini Comb design was modified by adjusting the inner diameter of the twistable cover to fit each

system. Figure S2 shows a top and bottom view of the Mini Comb when incorporated into the Artinis (left), ETG 4000 (middle) and NIRScout (right) fNIRS systems.

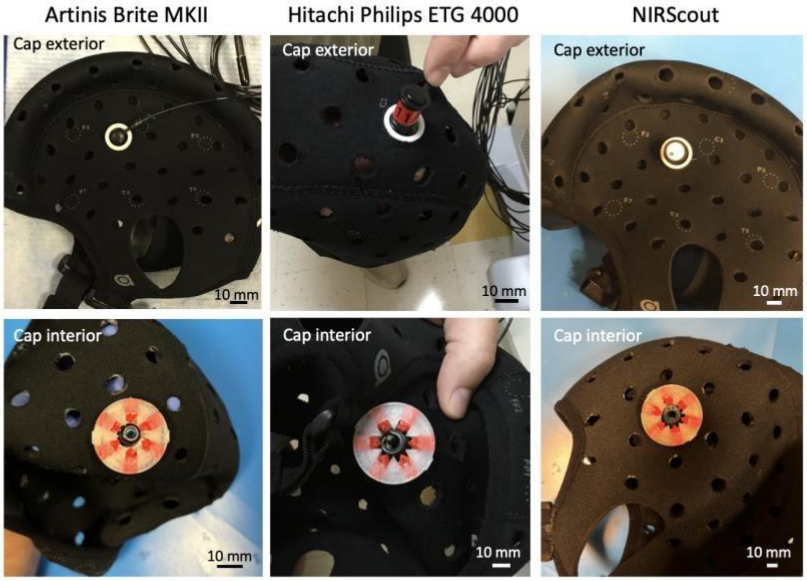

Figure S2. Mini Comb incorporated into two commercial systems. From left to right, the images show the Mini Comb in an open configuration containing the optodes of the Artinis Brite MKII, Hitachi Philips ETG 4000, and NIRScout system optodes.

In addition to modifying the inner diameter of the top of the twistable cover, the Mini Comb design can be modified across numerous other parameters. To accommodate differing optode lengths, the height of each individual piece can be modified to allow for proper coupling between the optode and scalp. Currently, the Mini Comb can be used to measure signal across channels greater than 24 mm in length, but to accommodate channels of shorter separation distance, the outer diameter of the support piece (currently measuring 24 mm as shown in Figure 1b) can be adjusted.

Because the Mini Combs need to be compatible with (i.e., not obstruct or cause damage to) electronics components present in all fNIRS systems, we chose to make them out of polylactic acid (PLA). PLA can be easily cleaned and disinfected, is durable enough to be used repeatedly, and it can be constructed quickly via 3D printing. The design also needed to make comfortable contact with the participant's scalp. Comb-like extrusions that are too sharp or rigid could damage or scratch to the scalp[25-29]. Additionally, application of pressure to the scalp could cause hair loss or alopecia[30-31]. Because some fNIRS imaging procedures can span multiple hours in duration, this prolonged pressure is a significant concern. To address these concerns, we developed a soft interface for the comb features. The soft interface was formed by coating the extrusions with an approximately one-mm-thick layer of Dragon Skin FX-Pro silicone, which is a soft, skin- and biosafe silicone rubber.

1.3 Wigged mannequin population and results from hair characterization

To create the wigged mannequin population, we utilized one bald mannequin head and ten wigs (Figure S3). All wigs were supplied with a netting headcap that was placed on the mannequin first. Each wig was then placed on the mannequin's head by attaching a clip on the wig to the netting headcap and covering the entire netting area. To ensure the wig was firmly on the mannequin, we gently shook the mannequin head and observed for any moment in the wig location. If movement occurred, we reattached the wig to the netting and repeated the process. The hair characteristics of each wigged mannequin were determined by the characterization process previously mentioned across three locations (C3, Cz, and C4) standardized by the 10-10 international optode layout. The hair length, shaft thickness, curl/coil, and density measurements were averaged across the three locations to account for imperfections in the wigs.

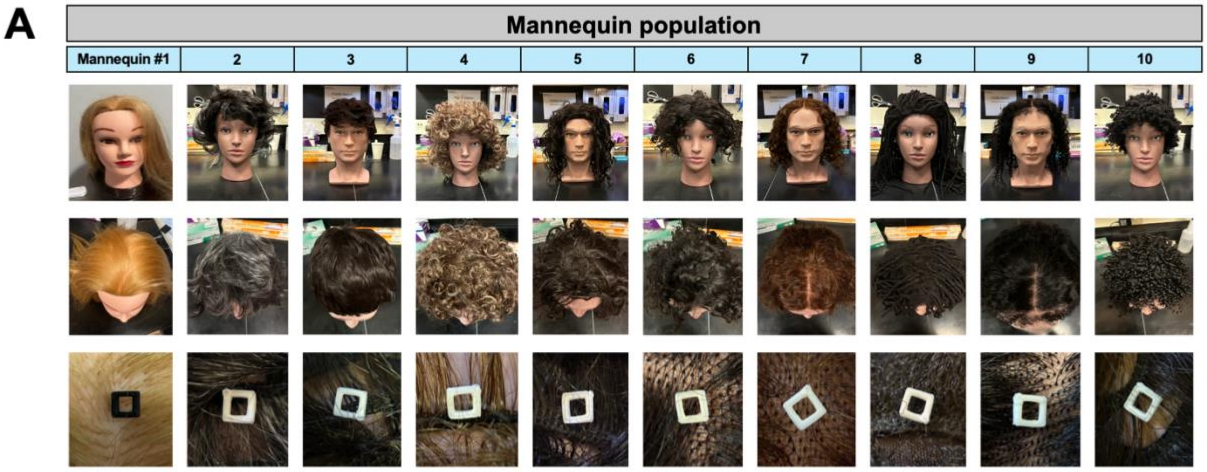

B

Mannequin hair characterization results

Mannequin #	1	2	3	4	5	6	7	8	9	10
Length (cm)	32.6	10.2	7.56	31.3	23.7	42.6	21.2	18.4	38.7	14.1
Shaft thickness (mm)	0.073	0.086	0.098	0.101	0.092	0.108	0.083	0.097	0.079	0.087
Curl or coil	1A	2A	2B	2C	3A	3B	3C	4A	4B	4C
Density (hairs/mm^2)	1.22	1.56	2.11	1.89	2.11	1.44	1.56	1.89	2.44	3.11
Color	8	2	4	6	2	2	6	3	1	1

Figure S3. (a) Images of the wigged mannequin population. (b) Results from wigged mannequin hair characterization.

At each location, three clearance trials were performed, and the cleared hair was photographed and passed through a Matlab script used to quantify the amount of clearance created. Between the three individual clearance trials, the hair that was cleared was adjusted to mimic the initial setting of the wig to ensure that no clearance effects remained for future trials.

The full wigged mannequin population is pictured in Figure S3A. The results from hair characterization on the wigged mannequins appear in Figure S3B. The tested wigged mannequins covered a wide spectrum of hair characteristic combinations, as shown by the values in the table. Wigged mannequins also varied in hair color, though hair color was not a significant factor in hair clearance on mannequins.

Post hoc, we tested two additional wigged mannequins to (1) ensure that at least one loose-haired mannequin was tested for each hair type and (2) observe how hair clearance is affected when hair type is held constant and other hair characteristics are allowed to change. The two mannequins tested had Type 4A hair and are shown in Figure S4. Using the same hair clearance procedure as described for the original ten wigged mannequins, we found that the optimal sliding leg design differed for each of the three Type 4A wigs. The optimal sliding leg design pairs were Design F for Mannequin 8, Design H for Mannequin 11, and Design G for Mannequin 12. These results support the point detailed in the Discussion section that a more complex model should be used to properly match sliding leg designs with hair populations, whether mannequin or human, to create the optimal amount of clearance. Based on the results shown in Figure S4C, both hair density and strand thickness should more heavily influence sliding leg design selection in future studies.

A

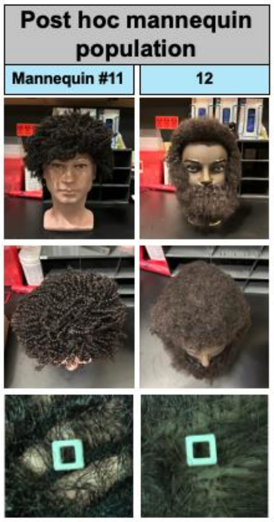

B

Post hoc hair characterization results

Mannequin #	11	12
Length (cm)	20.1	12.7
Shaft thickness (mm)	0.063	0.102
Curl or coil	4A	4A
Density (hairs/mm^2)	2.66	1.22
Color	1	4

C

Post hoc hair clearance results (N = 9) — Highest (51.7%) / Lowest (22.4%)

Sliding leg design	Mannequin Curl/Coil	4A (Mannequin 11)	4A (Mannequin 12)
	Design A	39.1%	47.6%
	Design B	28.4%	43.5%
	Design C	22.4%	39.1%
	Design D	29.5%	42.8%
	Design E	36.6%	50.2%
	Design F	37.6%	48.6%
	Design G	41.2%	51.7%
	Design H	41.5%	47.2%

Figure S4. (a) Images of the two Type 4A wigged mannequins tested post hoc. (b) Results from post hoc hair characterization. (c) Results from the post hoc hair clearance that follows the procedure outlined in the Mannequin Testing section of the manuscript.

1.4 Mannequin testing hair clearance quantification

The images used to quantify hair clearance in the wigged mannequin population were captured with the 8-megapixel main camera on the Samsung Galaxy Tab A7 Lite and was cropped so that only the 10.3-mm circular area cleared by the Mini Comb was visible. The images were then uploaded to the script that displayed the image and prompted the user to interactively select five pixels on the image that represented the scalp. The images were then converted to grayscale, and the grayscale intensity values at the selected scalp pixels were compiled. The largest intensity value of these pixels then became the intensity threshold in the script, meaning that any pixel with intensity greater than this value was considered hair and any pixel with intensity lower than this value was considered scalp. Each pixel in the image was then binarily identified as either hair strand or scalp. The number of nonzero pixels, which represent scalp, was used to calculate the percent of hair clearance achieved. The pipeline of the Matlab script is contained in Figure S5.

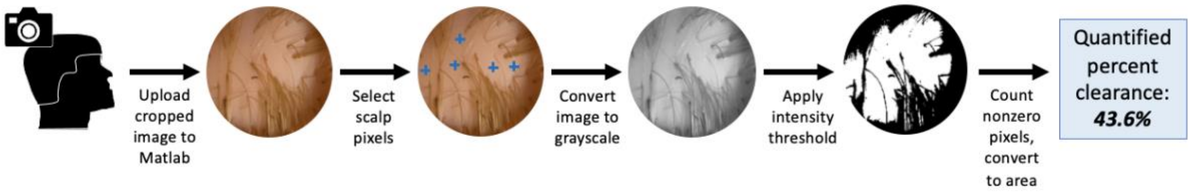

Figure S5. Overview of Matlab script used to quantify hair clearance. Blue '+' icons represent points selected by the user during image processing."
